# Response of soil structure and crop yield to soft rock in Mu Us sandy land, China

**DOI:** 10.1038/s41598-022-04860-5

**Published:** 2022-01-18

**Authors:** Jian Zhang, Zhen Guo

**Affiliations:** 1 School of Water and Environment, Chang’an University, Xi’an, 710054 China; 2 Shaanxi Provincial Land Engineering Construction Group Co., Ltd., Xi’an, 710075 China

**Keywords:** Civil engineering, Solid Earth sciences

## Abstract

The sandy land leaks water and fertilizer, and is seriously degraded, while the soft rock has a special depression structure, which plays a role in retaining water and fertilizer. The application of soft rock new material to sand reclamation can improve the ecological environment and ensure the quality of basic cultivated land. The soft rock and sand were mixed in different volume ratios (1:0, 11:1, 5:1, 4:1, 3:1, 2:1, 7:5, 1:1, 5:7, 1:2, 1:3, 1:4, 1:5, 1:11, 0:1) to prepare the composite soil, and its Raman spectrum characteristics, microstructure, texture composition and potato yield were studied. The results show that there are more silt and clay particles in the soft rock and more coarse particles in the sand. The peak position of the sand is 464.5 cm^−1^. With the increase of the content of the soft rock, the peak position decreases gradually. When the content of the soft rock accounts for more than 50%, the soil structure collapses and also becomes compact, at the same time the compressive stress is generated between the soil particles. When the ratio of soft rock to sand is 1:1, the soil texture is loam. The potato yield of the soil with the ratio of 1:5 of soft rock and sand cover increases significantly by 4.89–37.31% and 4.08–35.95% compared with that of 1:1 and 1:2 compound soils. Under the condition of 1:1 and 1:2 compounded ratio of soft rock and sand, there are more cementitious materials between the soil particles generated. The compounded ratio 1:5 is most suitable ratio for potato growth of local economic crop. The results confirmed that the Raman spectroscopy characteristics of SiO_2_ molecules can be used to study the cementation force between composite soil particles. When the compound ratio is 1:5, the soil improvement of Mu Us sandy land and the high yield of potatoes can be achieved, which could also provides a theoretical basis for sandy land remediation.

## Introduction

The Mu Us sandy land is located in the northwestern part of Shaanxi province of China, the soft rock and sand in the area are widely distributed^1^. The soft rock is as hard as stones, but it will become soft and muddy when mixed with water, while sand is loosely structured and loses more water and fertilizer^2^. Land desertification and soil erosion of soft rock are called "two hazards", which seriously restrict the sustainable development of the region and have become a focus of world economic development today^3^. The intensification of land desertification is one of the main ecological disasters, so it is necessary to strengthen agricultural scientific research in the desertification area^4^. Therefore, exploring the way to increase the new cultivated land, and improving the arable land area and crop yield in the desertification area are beneficial to promote the sustainable development of agriculture in our country.

Up to now, the research on the soft rock has mainly focused on the nature, species and distribution of the soft rock, the characteristics of soil and water loss and vegetation management, ecological security evaluation, etc.^5,6^, however, the research on the resource utilization of soft rock is rare. The study on sandy land management mainly focuses on prevention and controlling, including vegetation, engineering and chemical measures, such as vegetation restoration, shelterbelt construction, square sand barrier, chemical sand-fixing agent, etc. The main practice in the development and utilization of sandy land was the pull-transport loess mulching method^7,8^. Some scholars have proposed that the sand and soft rock have obvious complementary characteristics in particle size composition^9^. The cementation material of soft rock can effectively promote the mutual adsorption between particles and the formation of a solid structure^10^. Based on the understanding of the characteristics of the two substances, the author proposes to use the complementarity of the physical composition of the "two evils" to compound them into a new type of “soil”. Changing the "two evils" into "one treasure" to realize the resources utilization of soft rock and sand. In order to understand the mechanical properties of the composite soil, some scholars studied the microstructure of the composite soil by scanning electron microscopy, which showed that the permeability of the composite soil was greatly reduced due to the optimization of its grain grading^11^. As the proportion of soft rock increased, the soil particles changed from point contact to surface contact, and the adhesion on the surface of the particles gradually increased^12^. Wang et al.^13^analyzed the hydraulic characteristics of the sandy soil in the Mu Us Sandy Land, and the results showed that the addition of soft rock can significantly increase the bulk density of the sandy soil with reducing the saturated water content and saturated hydraulic conductivity. The study on the composite soil of soft rock and sand mainly revealed the physical reconstruction phenomenon, but how the colloids in soft rock play a mechanical protection mechanism in sandy land is still unknown.

In this study, the range of the ratio of soft rock and sand was analyzed by laboratory test, then the representative mixture ratio was selected for field plot experiment, and then extended to a large area at the later stage. In order to improve and maintain the quality of farmland formed by the combination of soft rock and sand, and to realize the diversified application of the compound soil, it is necessary to understand the microstructure of the compound soil to reflect the mechanical properties of the soil. It can provide a theoretical basis for the formation mechanism of soft rock and sand compound soil. The objectives of this study are to (1) reveal the influence of different proportions of soft rock on the mechanical composition and texture of sandy soil; and (2) screen the ratio which is the most suitable one for the growth of local crops, conduct field trials and study the microstructure of the composite soil. Three hypotheses are put forward: (1) when the ratio of soft rock to sand is 1:1, the critical point of soil texture change show ups and the peak position of Raman spectra changes abruptly; (2) when the ratio is 1:5, it is most suitable ratio for the growth of local crops; and (3) after 3 years of planting, the soil with a ratio of 1:1 has more adhesives and its structure was tighter.

## Results and discussion

### Particle composition and microstructure of soft rock and sand

The mass fraction of coarse sand grains in the soft rock is 24.52%, which is about 1/4 of the coarse sand grains in the sand. The mass fraction of the silt grain and clay grain in the soft rock is 64.98% and 10.50%, respectively, which is about 16 times and 20 times as much as the mass fraction of the silt grain and clay grain in the sand (Table 1). The results of Cheng et al.^14^ study were consistent with this study. Therefore, compared with sand, the content of large particles in soft rock is low, but the content of medium and small particles is high. Wang et al.^6^ research results also believed that soft rock contains more clay and silt particles.

**Table 1:** Particle composition of soft rock and sand.

Material	Coarse sand grain ratio (%)	Silt grain ratio (%)	Clay grain ratio (%)
	0.05–2 mm	0.002–0.05 mm	< 0.002 mm
Soft rock	24.52	64.98	10.50
Sand	95.37	4.10	0.53

**Figure 1:**
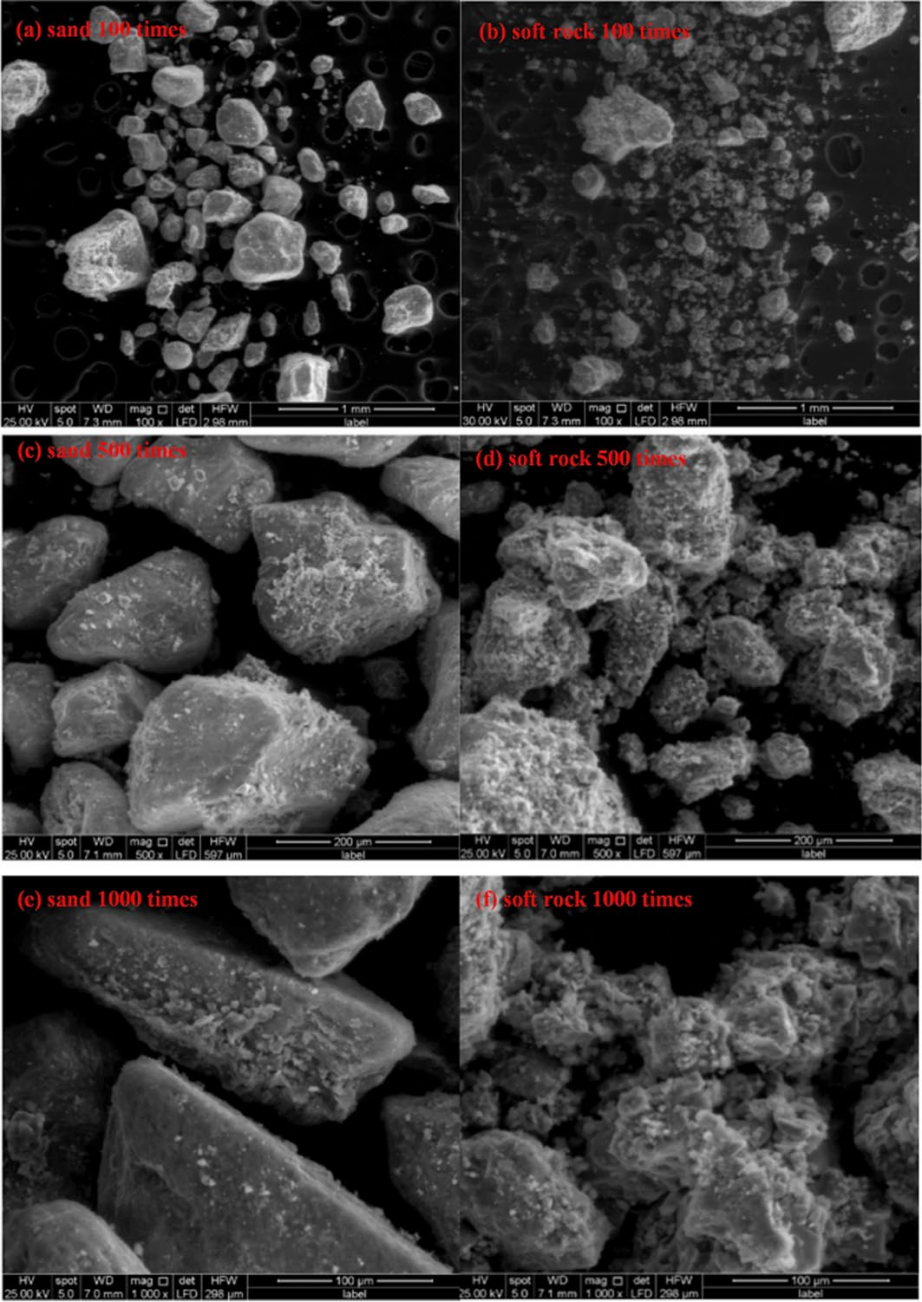
Scanning electron microscope images of sand and soft rock.

The result is shown in Fig. 1a and Fig. 1b were microscopic images of sand and soft rock at 100 times magnification. It can be seen that there are many large particles in sand, but more small and medium-sized particles in the soft rock than in the sand. The soft rock and sand have certain complementarity to each other in grain composition. The combination of soft rock and sand can make up for the lack of fine particles to a certain extent^15^. Figure 1c,e are images of sand at 500 and 1000 multiples, and Fig. 1d,f were images of soft rock at 500 and 1000 multiples. It can be seen from the images of 500 and 1000 multiples that the surface of the sand particles was relatively smooth, the gap between the particles is large, and the degree of inter-particle bonding is weak; and the surface of grain of soft rock is relatively rough because of the attachment and other reasons, the distance between particles is relatively short, and the connection between particles is relatively close. The results are similar to those of Chen^16^ and Liu et al.^17^. It can been seen that the addition of soft rock was beneficial to improve the loose structure of sandy land and promote soil agglomeration and cementation. Wang et al.^13^ pointed out that the addition of soft rock to sandy soil will increase the agglomeration of sandy soil, mainly due to the increase in the mass fraction of water-stable aggregates larger than 0.25 mm.

### Raman spectra effect of different ratios of soft rock and sand

**Figure 2:**
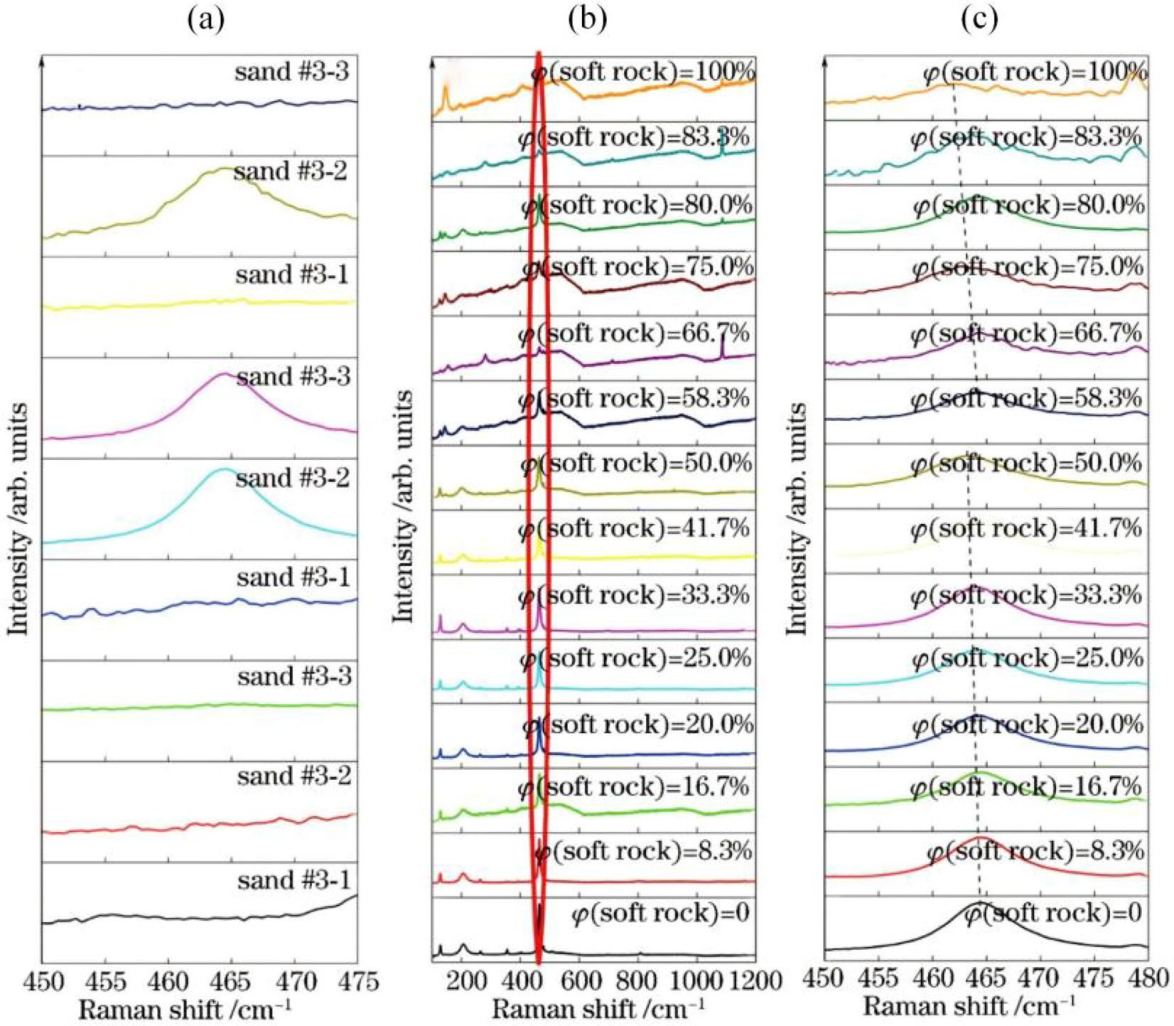
Raman spectra of soft rock, sand and composite soil. (**a**) is a magnified view of nine Raman spectra of sand; (**b**) and (**c**) are the Raman spectra of the soft rock and the composite soil and their magnified images.

It can be seen from Fig. 2a that among the nine Raman spectra lines of sand, six of which cover the Raman signal because of the strong fluorescence background, and the other three peaks have very high repeatability, and the peak positions of the three peaks are almost unchanged. It can be seen from Fig. 2b that a characteristic peak appears at the Raman shift of 146 cm^−1^ and 464 cm^−1^, in which the peak at 464 cm^−1^ is the peak of SiO_2_. The sand has a characteristic peak at 128, 206, 264, 355, 394, 403, 464, 809, 1082 and 1160 cm^−1^, respectively. These peaks are attributed to different vibrational modes of SiO_2_ molecules^18–20^. This is because the highest chemical content in soft rock and sand is SiO_2_. The mass fraction of SiO_2_ in the soft rock is more than 60%, and the content of SiO_2_ in the sand is higher than that in the soft rock^13^. The characteristic peaks of SiO_2_ also appear in the Raman spectra of the composite soil, in which the peak at 464 cm^−1^ is the strongest characteristic peak of SiO_2_, and Fig. 2c shows the magnification of the peak.

**Figure 3:**
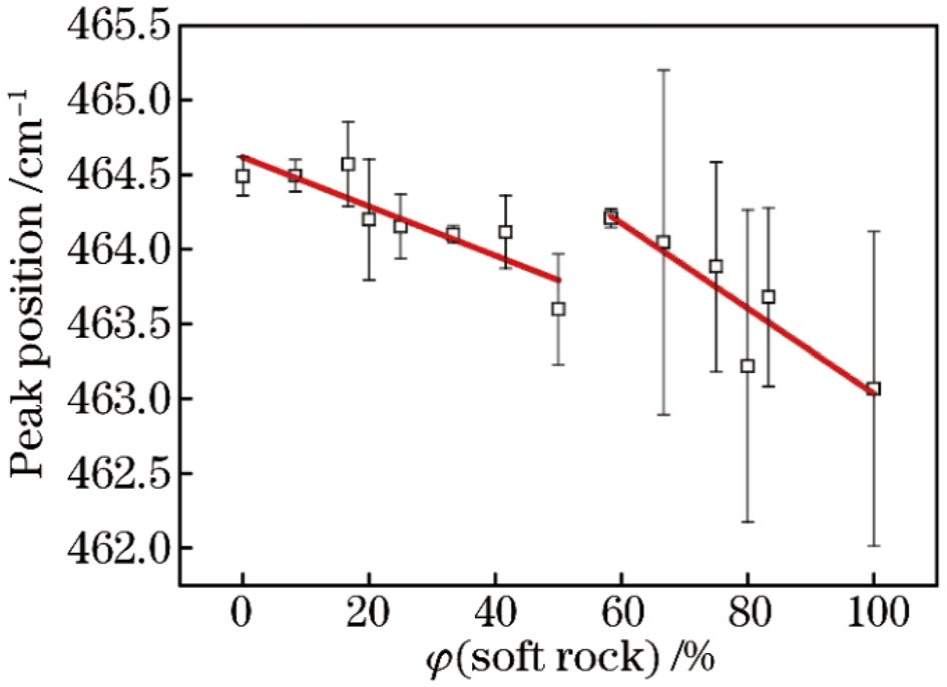
Peak position at Raman shift 464 cm^−1^ of SO_2_ in composite soil varying with content of soft rock.

It can be seen from Fig. 3 that with the increase of soft rock content in the sand, the characteristic peak position gradually reduces. When the content of soft rock begins to be greater than that of sand, the characteristic peak position suddenly increases, and then the characteristic peak position gradually decreases with the increase of the soft rock content. The shift of the peak position of the Raman spectra may be caused by doping or stress^21,22^. Tang et al.^23^ based on Raman spectrum analysis showed that after the external force was applied to the sample, the Raman peak of Si shifted blue with the increase of pressure, indicating that the external pressure caused compressive stress in the material. Therefore, the stress type corresponding to the peak position red shift (or blue shift) in this study is tensile stress (or compressive stress). According to the peak position shift in the Raman spectrum, the mechanical mechanism of the sample can be inferred.

### Analysis of mechanical properties of soft rock and sand compound soil

**Figure 4:**
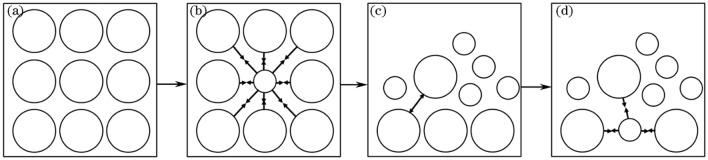
Evolution diagram of cementation of compound soil particles.

The mechanical mechanism of the sample can be inferred based on the shift of the peak position in the Raman spectra (Fig. 2, Fig. 3). With the increase of the content of soft rock in the compound soil, the cementation environment of the soil particles change^24,25^. As a result, the distance between the large particles and the small particles in the sand is larger than that between the large particles and the large particles in the sand, so that the particles are drawn together to produce tensile stress, as shown in Fig. 4(a) and Fig. 4(b). This is because the soft rock contains more medium and small particles, which occupy the position of some large particles in the sand (Table 1) . When the volume fraction of the soft rock in the compound soil is more than 50%, there are many small particles in the compound soil, and the large particles are not enough to support the structure of the soil, so the soft rock collapses, and the size of the soil particles is suddenly compacted. The soil grains are squeezed each other, resulting in compressive stress, as shown in Fig. 4(c), and the peak position in Fig. 3 increases abruptly. With the further increase of the content of the soft rock, the soil structure tends to be stable. The content of small particles in the compound soil was getting higher and higher, and the bonding and mutual polymerization forces are strong, so the particles are pulled each other, which leads to the stretching effect is continuously enhanced, as shown in Fig. 4(d). The peak position of the right half of the corresponded Fig. 3 decreases again. When the compound soil was all soft rock, the peak position decreases to 463.1 cm^−1^. Zhang et al.^26^ shown that the clay of soft rock has colloidal properties, interaction between soft rock and sand usually include van der Waals force, coulomb force, electric double layer potential energy and hydration force, etc. After the soft rock was mixed with the sand, the distance between the clay particles in the soft rock and the sand particles in the sandy soil was shortened under the action of physical precipitation, and the force between particles increased^26^, which was consistent with the results of this study.

### Mechanical composition of compound soil

Table 2 shows the mechanical composition and texture distribution of the compound soil. It can be seen that with the decrease of the content of the soft rock, the texture of the compound soil changes from sandy soil to silty loam. When the coverage ratio of soft rock and sand is 1:1, it is the boundary point of sandy loam and silty loam. When the content of soft rock in the compound soil exceeds 50%, due to the silt grains is more abundant, the soil evapotranspiration is larger, and the soil water storage, which leads to the results that water guiding and water retention functions are weakened, which is not conducive to crop growth^27^. Among them, the mechanical properties of the compound soil also verified the adverse effects of the soil structure when the content of soft rock exceeds 50% (Fig. 4). Cheng et al.^28^ point out that the finer the soil particles, the higher the volume fractal dimension. With the increase of planting years, the texture of 1:1 compound soil changed most significantly, similar to the results of this study^28^. Wang et al.^2^ pointed out that when the proportion of soft rock was less than 50%, the soil texture was silt loam, which was conducive to crop growth.

**Table 2:** Mechanical composition of different proportions of compound soil.

Particle size ratio (%)	Mixed ratio (soft rock:sand)											
	5:1	4:1	3:1	2:1	7:5	1:1	5:7	1:2	1:3	1:4	1:5	1:11
Sand	26.61	28.92	31.30	33.76	40.30	46.84	55.76	64.67	69.77	74.79	84.25	92.63
Silt	64.18	62.28	59.62	58.58	51.75	44.92	36.85	30.04	25.03	20.08	10.16	5.01
Clay	9.21	8.80	9.08	7.66	7.95	8.24	7.39	5.29	5.20	5.13	5.59	2.63
Texture	Sandy loam					Loam	Silty loam					

### Potato yield in soft rock and sand compound soil

**Figure 5:**
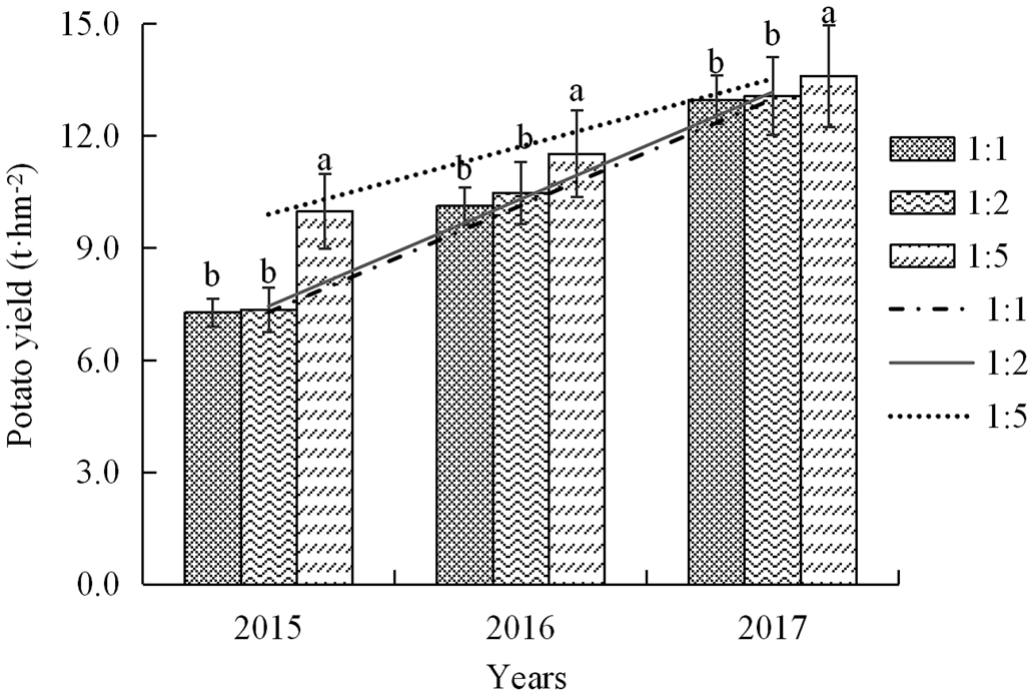
Potato yield in compound soil under different planting years. Lowercase letters indicate differences in 5% levels of different compound soils under the same planting years.

According to the analysis of Raman spectra effect and mechanical properties (Figs. 2, 3, 4), when the content of soft rock in the composite soil exceeds 50%, which is not suitable for local planting of potato and other rhizome economic crops^29,30^. Therefore, the experiment of potato planting was carried out in this study, in which the ratio of soft rock and sand was 1:5, 1:2 and 1:1, respectively. The results of three consecutive years of planting in 2015 show that potato yields increase linearly with years of growth, and potato yields decrease with the increase soft rock content in the compound soil (Fig. 5). In the past three years, the yield of potato with soft rock and sand ratio of 1:5 soil was 9.98 t·hm^−2^, 11.52 t·hm^−2^ and 13.61 t·hm^−2^, respectively. Compared with 1:1 and 1:2 compound soil, the potato yield in the soil increases significantly by 4.89–37.31% and 4.08–35.95%, and the potato yield in the 1:2 compound soil is 0.77–3.4%, higher than that of the 1:1 soil. Sun et al.^31^ research has the same result. It can be seen that the soil with 1:5 ratio of soft rock and sand can promote the high yield of potato.

### Microstructure of soft rock and sand compound soil under different magnification

**Figure 6:**
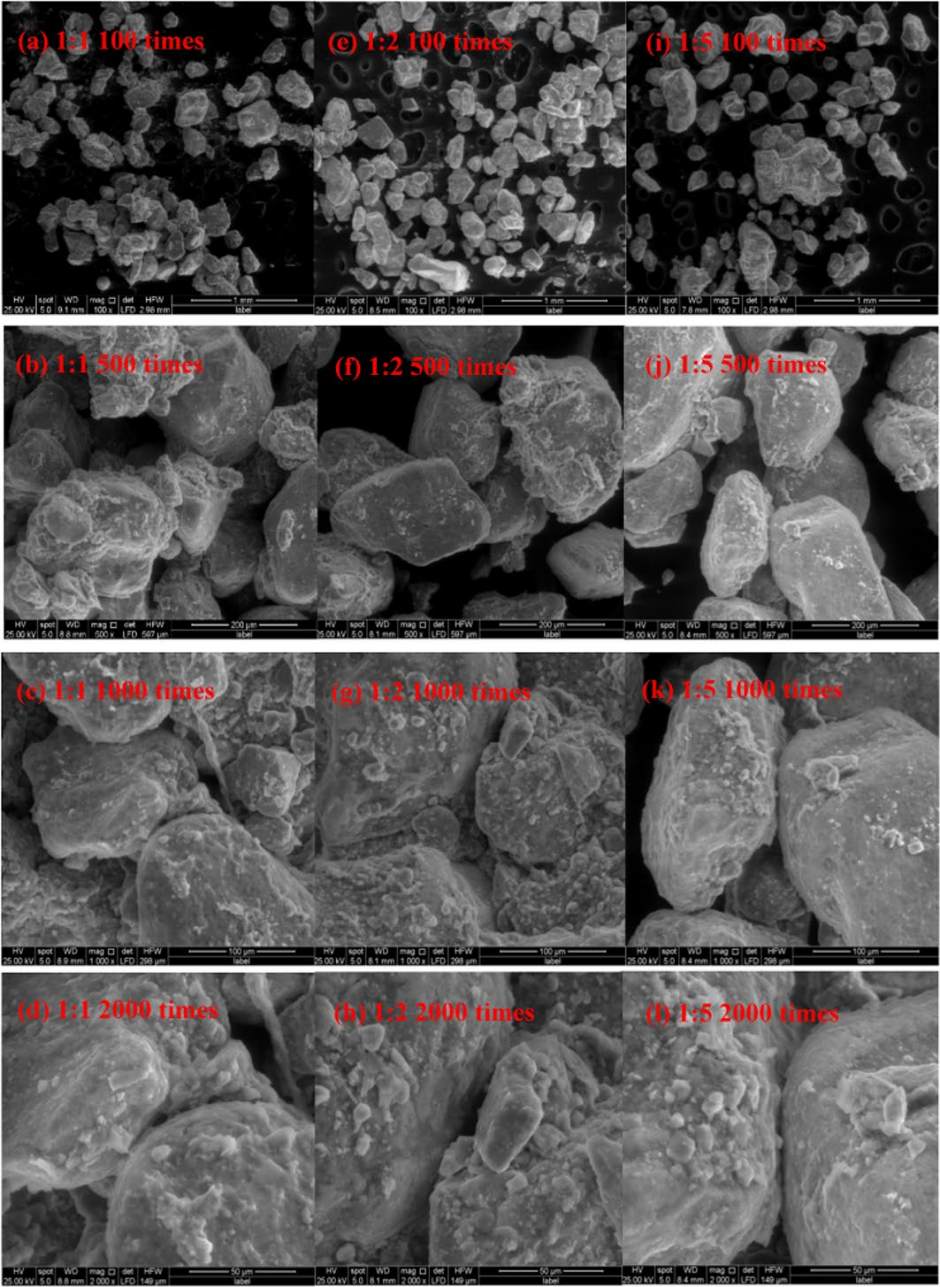
Scanning electron microscopy images of different proportions of compound soil after 3 years of planting. (**a**–**d**), (**e**–**h**), (**i**–**l**) are images of soft rock and sand compound ratios of 1:1, 1:2, 1:5, respectively; (**a**, **e**, **i**), (**b**, **f**, **j**), (**c**, **g**, **k**), (**d**, **h**, **l**) are microscopic images of magnifications of 100, 500, 1000, 2000 times, respectively.

It can be seen from the low magnifications (100 and 500 times) images that there are more cementitious substances between the particles in the compound soil at 1:1 (Fig. 6). With the increase of the content of sand, the particles become dispersed and the cementitious substances gradually decrease. In the high-magnification images (1000 and 2000 times), it can be seen that there are more attachments on the surface of soil particles under the conditions of 1:1 and 1:2 compound soil than in the case of more sand content at 1:5. Moreover, the particles are mostly in surface contact, and there are more cement materials between the particles to bond them well, and a certain pore is retained while the soil has good agglomeration. On the basis of Wang’s previous field plot experiment^6^, through large-scale planting in the compound soil, the results showed that when the volume ratio of the soft rock to the sand was 1:2 (the soft rock volume fraction was 33.3% in the compound soil), it was suitable for planting corn. When the volume ratio of the soft rock to the sand was 1:5 (the soft rock volume fraction was 16.7% in the compound soil) it was still suitable for planting potatoes.

## Conclusions

The soil with the ratio of 1:5 of soft rock and sand could promote the high yield of potato, which can be widely popularized in Mu Us sandy land. The particle compositions of soft rock and sand are complementary. It is beneficial to improve the loose structure of the sandy land and promote the soil agglomeration and cementation by compounding the soft rock and sand. With the increase of the content of the soft rock, the texture of the compound soil will change from silty loam, loam to sandy loam. Under the conditions of 1:1 and 1:2 between the soft rock and sand, there are a lot of cementitious materials between the soil particles and the particles, which can keep a certain pore and the soil has a good cohesion at the same time. Therefore, in the later stage, according to the growing demanding of different crops in different regions, the engineering cost should be considered comprehensively, and different compound ratios should be chosen according to the specific situation. This research provides a broad path and technical support for ensuring food security and the sustainable development of sandy land.

## Materials and methods

### Study site

The soft rock and sand used in this study were collected from Xiaojihan Township (109°58′E, 38°44′N) in Yuyang District, Yulin City, Shaanxi Province, China. The area was located at the southern edge of the Mu Us sandy land and the northern end of the Loess Plateau, composed of gentle sand dunes and sand chains. The region is affected by the temperate continental monsoon climate with obvious seasonal changes. The average annual precipitation was 413.9 mm, the average annual temperature was 8.1 °C, the average frost-free period was 155 days, and the average annual evaporation was 1904 mm. Most of the area was deserted, not only forest,the soil type mainly aeolian sandy soil (sand), with only wormwood growing sporadically.

### Experiment design

The soft rock is of low diagenesis, it is hard as stone when dry and soft, but it will become mud when exposed to water^32,33^. Sand has large porosity, poor cementation, low structural strength, and poor water and fertilizer retention^34^. The minerals in the soft rock mainly contain quartz, montmorillonite, feldspar, calcite, illite, kaolinite and dolomite^35^. The main chemical components of soft rock were SiO_2_, Al_2_O_3_, Fe_2_O_3_ and CaO^36^. The minerals in sand are mainly quartz (SiO_2_), and the other minerals are mainly feldspar, kaolinite, calcite and amphibole^3^. The collected soft rock and sand were respectively ground and sieved with a pore size of 1 mm, and then the sieved powder was weighed and mixed according to the 15 volume ratio shown in Table 3 (1:0, 11:1, 5:1, 4:1, 3:1, 2:1, 7:5, 1:1, 5:7, 1:2, 1:3, 1:4, 1:5, 1:11, 0:1), and each group was repeatedly prepared three times. According to the movement of peak position, the change of mechanical properties, the adaptability of local potato planting and the growth characteristics of potato, fiveteen groups of compound soil were analyzed by Raman spectra and mechanical composition analysis. The mixed volume of loose texture was selected for the field plot experiment.

**Table 3:** Proportion of soft rock and sand in compound soil.

Sample number	Volume fraction of soft rock(%)	Volume ratio of soft rock to sand
1	100	1:0
2	91.7	11:1
3	83.3	5:1
4	80.0	4:1
5	75.0	3:1
6	66.7	2:1
7	58.3	7:5
8	50.0	1:1
9	41.7	5:7
10	33.3	1:2
11	25.0	1:3
12	20.0	1:4
13	16.7	1:5
14	8.3	1:11
15	0	0:1

The field test plot was to simulate the land condition of the mixed layer of soft rock and sand in the Mu Us sandy land. The experimental plot was to lay a mixture of soft rock and sand at 0–30 cm. The selected ratio of soft rock to sand (1:1, 1:2, 1:5) was repeated for three times in the experimental field with an area of 15 m × 12 m = 180 m^2^. The field trial implements a potato cropping system once a year, planted in mid-April and harvested in mid-to-late September each year. Artificial planting mode is adopted through the year. The test fertilizer types in the test field were urea, diammonium phosphate and potassium chloride. The fertilizer application amount was N 300 kg·hm^−2^, P_2_O_5_ 375 kg·hm^−2^ and K_2_O 180 kg·hm^−2^ per year. All phosphate fertilizers and potash fertilizers are used as base fertilizers, and 50% of nitrogen fertilizers are used as base fertilizers. One to two days before planting, weigh the three kinds of fertilizers according to the required amount of each plot and mix them evenly, sprinkle them evenly on the soil surface, and then properly rake the fertilizer to mix the topsoil. The remaining 50% of the nitrogen fertilizer is topdressed at the potato seedling stage and after flowering.

### Soil sample collection

The mixture was stirred until uniform, and then dried at 100 °C for about 3 h. The dried compound soil samples were ground again and after sieved with a pore size of 1 mm, they were placed in an aluminum box for Raman spectra analysis and mechanical composition analysis^1^. Initial samples were not repeated. In May 2015, the field plot experiment started and the potatoes have been planted continuously for 3 years. After the potato were harvested in October 2017, 0–30 cm soil samples of were collected from each plot, and three mixed soil samples were collected in each plot as a replicate. Each mixed sample was formed by mixing samples of five points collected uniformly. The collected soil samples were removed from animal and plant residues and passed through a 1 mm sieve for scanning electron microscopy analysis.

### Experiment method

The prepared 15 sets of powder samples were tested using an microscopic confocal Raman spectra (HR800, HORIBA JobinYvon, France). During the test, the samples from the aluminum box from top to bottom was taken with a spoon, spread them evenly on the slide, and then gently flattened them with another slide. The slide carrying the sample was placed on the platform and tested at three different points of each sample. Since the compound soil of each volume ratio was repeatedly prepared three times, nine Raman lines were measured for each volume ratio soil sample. Using a laser with a wavelength of 532 nm, the Ramanspectroscopy was measured with a telephoto lens with a magnification of 50, a numerical aperture of 0.35, and a field of view of 26.5. The laser power reached the surface of the sample was about 10 MW,the grating constant was 1800 mm^−1^, and the resolution was about 0.5 cm^−1^.

After the soil sample collected in the field plot was treated, it was solidified by epoxy resin, coarsely ground, artificially ground and polished by a sander to make the surface flat and smooth. The micro samples with a diameter of 5 mm and a height of 3 mm were obtained. After they were washed with distilled water, the residual water on the surface was removed. The dried sample was subjected to gold plating, using a scanning electron microscope (Q45, FEI, USA) at a voltage of 15 kV, and scanned in an “S” type with magnifications of 100, 500, 1000, and 2000.

The particle sizes of sand and soft rock were measured by Mastersizer 3000 laser particle size analyzer (Mastersizer 3000, Malvern Panakko, UK). All samples were taken 10 g, and each soil sample was repeatedly tested 3 times. After the potatoes were harvested, 40 potato plants were collected by diagonal method for fresh sample measurement.

### Data analysis

The Nanomeasure software was used to measure the particle size. The results and output data were statistically analyzed by Excel 2016 (Microsoft, USA) and plotted by Origin 8.5 (OriginLab, USA).

### Research involving plants

Experimental research and feld studies on potato plants were complied with relevant institutional guidelines.
